# Equine osteoarthritis modifies fatty acid signatures in synovial fluid and its extracellular vesicles

**DOI:** 10.1186/s13075-023-02998-9

**Published:** 2023-03-09

**Authors:** Anne-Mari Mustonen, Nina Lehmonen, Tommi Paakkonen, Marja Raekallio, Reijo Käkelä, Tytti Niemelä, Anna Mykkänen, Sanna P. Sihvo, Petteri Nieminen

**Affiliations:** 1 Institute of Biomedicine, School of Medicine, Faculty of Health Sciences, University of Eastern Finland, P.O. Box 1627, FI-70211 Kuopio, Finland; 2 Department of Environmental and Biological Sciences, Faculty of Science, Forestry and Technology, University of Eastern Finland, P.O. Box 111, FI-80101 Joensuu, Finland; 3 Department of Equine and Small Animal Medicine, Faculty of Veterinary Medicine, University of Helsinki, P.O. Box 57, FI-00014 Helsinki, Finland; 4 Molecular and Integrative Biosciences Research Programme, Faculty of Biological and Environmental Sciences, University of Helsinki, P.O. Box 65, FI-00014 Helsinki, Finland; 5 Helsinki University Lipidomics Unit (HiLIPID), Helsinki Institute of Life Science (HiLIFE) and Biocenter Finland, P.O. Box 65, FI-00014 Helsinki, Finland

**Keywords:** Extracellular vesicle, Fatty acid, Horse, Joint disease, Osteoarthritis, Synovial fluid

## Abstract

**Background:**

Individual fatty acids (FAs) and their derivatives (lipid mediators) with pro-inflammatory or dual anti-inflammatory and pro-resolving properties have potential to influence the health of joint tissues. Osteoarthritis (OA) is an age-associated chronic joint disease that can be featured with altered FA composition in the synovial fluid (SF) of human patients. The counts and cargo of extracellular vesicles (EVs), membrane-bound particles released by synovial joint cells and transporting bioactive lipids, can also be modified by OA. The detailed FA signatures of SF and its EVs have remained unexplored in the horse — a well-recognized veterinary model for OA research.

**Methods:**

The aim of the present study was to compare the FA profiles in equine SF and its ultracentrifuged EV fraction between control, contralateral, and OA metacarpophalangeal joints (*n* = 8/group). The FA profiles of total lipids were determined by gas chromatography and the data compared with univariate and multivariate analyses.

**Results:**

The data revealed distinct FA profiles in SF and its EV-enriched pellet that were modified by naturally occurring equine OA. Regarding SFs, linoleic acid (generalized linear model, *p* = 0.0006), myristic acid (*p* = 0.003), palmitoleic acid (*p* < 0.0005), and n-3/n-6 polyunsaturated FA ratio (*p* < 0.0005) were among the important variables that separated OA from control samples. In EV-enriched pellets, saturated FAs palmitic acid (*p* = 0.020), stearic acid (*p* = 0.002), and behenic acid (*p* = 0.003) indicated OA. The observed FA modifications are potentially detrimental and could contribute to inflammatory processes and cartilage degradation in OA.

**Conclusions:**

Equine OA joints can be distinguished from normal joints based on their FA signatures in SF and its EV-enriched pellet. Clarifying the roles of SF and EV FA compositions in the pathogenesis of OA and their potential as joint disease biomarkers and therapeutic targets warrants future studies.

**Supplementary Information:**

The online version contains supplementary material available at 10.1186/s13075-023-02998-9.

## Background

Individual fatty acids (FAs) and their derivatives, including classic eicosanoids and specialized pro-resolving mediators, have significant potential to influence the health of different joint tissues [1]. In humans, dietary saturated FAs (SFAs) can be associated with the development of osteoarthritis (OA), whereas polyunsaturated FAs (PUFAs) and monounsaturated FAs (MUFAs) may have opposite influence [2, 3]. Especially long-chain n-3 PUFAs, 20:5n-3 (eicosapentaenoic acid) and 22:6n-3 (docosahexaenoic acid) of marine origin, are considered beneficial due to their anti-inflammatory and chondroprotective effects [1]. However, there are inconclusive data about how efficiently FA supplements could affect the risk of OA, ameliorate pain, or enhance the function of OA joints in humans [4, 5]. The same applies to the horse [6, 7], a well-recognized veterinary model for OA research with important similarities to humans in respect to the thickness and bioproperties of articular cartilage [8]. Although n-3 PUFA supplements modify the synovial fluid (SF) FA profiles of horses, their effects on the markers of inflammation and cartilage degradation have been minor [7, 9]. However, 20:5n-3 and 22:6n-3 have reduced the expression of inflammatory factors (interleukins, cyclooxygenase-2) and cartilage-degrading proteinases and increased the synthesis of pro-resolving lipid mediators in equine synoviocyte cultures [10].

Extracellular vesicles (EVs) function as transport vehicles for FAs, other bioactive lipids, and their synthesis machinery between joint tissues [11]. They are released by most mammalian cells, including immune cells, chondrocytes, and synoviocytes, and can potentially induce both pro- and anti-OA effects. The EV lipid composition can influence the stability of EV membranes and the binding and uptake of EVs to target cells [12]. In human patients, OA SF is characterized by altered EV concentrations and cargo that may contribute to inflammatory processes, cartilage degradation, and pain [11]. The potential roles of EVs in synovitis and OA pathogenesis in the horse are just starting to be unravelled. In equine chondrocyte cultures, incubation with EVs from SF or mesenchymal stem cells (MSCs) can down-regulate genes involved in joint inflammation and cartilage degradation [13–15], and EVs from foetal bone marrow-derived cells can increase the survival of chondrocytes under inflammatory conditions [16]. In experimentally-induced synovitis, especially the levels of cluster of differentiation 44-positive EVs were elevated in horse SF [13].

Previous studies have investigated the phospholipid (PL) composition of equine SF [17, 18] but, to the best of our knowledge, the detailed FA signature (FAS) of SF and the effects of OA on FA profiles have remained unexplored in the horse. Regarding SF EVs, short-chain carboxylic acid *N*-modified phosphatidylserine molecules may be involved in experimentally-induced synovitis [13], while any precise information on the FAS of equine OA EVs is lacking. The aim of the present study was to compare the FA profiles (*i*) in SF and (*ii*) in its EV-enriched pellet between control, contralateral (CL), and OA joints of horses. We hypothesized that (*i*) naturally occurring equine OA would be characterized by elevated SFAs and n-6 PUFAs but reduced n-3 PUFAs in SF and that (*ii*) the FA profile of EV-enriched pellet would prove to be more inflammatory in OA.

## Materials and methods

### Animals and sampling

SF samples were obtained by arthrocentesis from meta-carpophalangeal (MCP) joints of 8 horses with OA and 8 horses without joint diseases at the Veterinary Teaching Hospital, University of Helsinki. Most of the horses were of warmblood breeds, but 1 Standardbred and 1 Estonian riding pony were also included. Informed owner consent was obtained for each animal, and ethical approval for SF collection and use was provided by the Viikki Campus Research Ethics Committee of the University of Helsinki (Statement 1/2018). The sampling was conducted immediately after medically-induced euthanasia (0.01–0.02 mg/kg detomidine hydrochloride, 0.01–0.02 mg/kg butorphanol tartrate, 0.05–0.1 mg/kg midazolam, 2.2 mg/kg ketamine hydrochloride, T-61 euthanasia solution) due to lameness or non-OA-related reasons including colic, back pain, leg injury, wound, sinusitis, neurological disease, and guttural pouch mycosis. The decisions to euthanize the animals had been made previously without any relation to the research protocol or sampling. The unprocessed SF samples were immediately frozen in liquid nitrogen and stored at –80°C until analysed. MCP OA was diagnosed *post-mortem* by experienced equine veterinarians based on the presence of wear lines, erosion of articular cartilage, and osteophytes. Joint surfaces were scored according to OA severity as follows: 0 = normal, 1 = mild OA, 2 = moderate OA, and 3 = severe OA [19]. SF samples were also harvested from CL MCP joints. Only one of them was classified as normal, and the rest had mild-to-moderate OA. In the *post-mortem* examination of control joints, the MCP joint surfaces either had no macroscopic abnormalities or revealed mild periarticular changes. Regarding medication, 3 control and 2 CL/OA horses had been treated with nonsteroidal anti-inflammatory drugs, 1 control and 1 CL/OA horse had received antibiotics, and 1 CL/OA horse had been treated with antifungals.

### Sample preparation and FA analysis

The FA profiles of the SF total lipids were determined with a previously described protocol [20]. Briefly, the unprocessed SF samples were transmethylated by heating with 1% H_2_SO_4_ in methanol (Fisher Scientific, Loughborough, UK) under N_2_ atmosphere, the formed FA methyl esters were extracted with *n*-hexane (Honeywell International Inc., Charlotte, NC, USA), followed by analysis by the Shimadzu GC-2010 Plus gas chromatograph (Shimadzu, Kyoto, Japan) with the flame ionization detector. The structures of FA methyl esters and dimethyl acetals (DMAs, derivatives of alkenyl chains from plasmalogen PLs) were confirmed by using electron impact mass spectra recorded by the Shimadzu GCMS-QP2010 Ultra with the mass selective detector.

For EV isolation, SFs were diluted 1:10 with sterile-filtered (pore size 0.22 μm) Dulbeccoʼs phosphate buffered saline (DPBS; Mediatech Inc., Manassas, VA, USA), centrifuged at 1000 × *g* for 10 min at +4°C, and the supernatants were centrifuged at 1200 × *g* for 20 min at +4°C. Finally, the supernatants were ultracentrifuged at <110,000 × *g* for 90 min at +4°C using the Beckman Optima L-90K ultracentrifuge with the 50.4 Ti rotor (Beckman Coulter Inc., Brea, CA, USA), and the obtained EV-enriched pellets were resuspended in 200 μl of sterile-filtered DPBS. Later, excess water was removed from the EV pellets by N_2_ stream followed by transmethylation and analysis as described above. Equine SF EVs have been characterized in our previous paper [19]. We documented two EV subpopulations, smaller EVs with a diameter of <100–200 nm and an average concentration of 1.8 × 10^10^ particles/ml, and larger EVs with a diameter of <1000–2000 nm.

The obtained chromatographic peaks were manually integrated with the Shimadzu GCsolution software. The gas chromatographic peak representing SF 22:6n-3 also included an unknown coeluting artefact, but this peak was regardless included in the analysis, as the artefact was not the major component of the peak. In EV-enriched pellets, the proportion of the artefact was significantly higher due to which this peak was left out of the manual integration of the EV samples. The results are expressed as mol-% in total lipid side chains in SFs or in ultracentrifuged EV-enriched pellets. The n-x abbreviations are used for the FAs, and the FA ratios and indices were calculated as previously outlined [20].

### Statistical analyses

Comparisons between the control, CL, and OA joints were performed with the generalized linear model (GLM) using the IBM SPSS *v*27 software (IBM, Armonk, NY, USA). As OA can be associated with ageing in the horse [21], age was used as a covariant in the analysis. The resulting *p* values were adjusted for multiple hypothesis testing controlling the false discovery rate by using the Benjamini–Hochberg procedure (Table 1; Table S1, S2). The Studentʼ t-test was utilized for comparisons between sample types (SF and EV-enriched pellet). The distribution of genders in the study groups was tested with the Fisher’s exact test, and correlations between FA proportions and age were calculated with the Spearman correlation coefficient (*r*_*s*_). The *p* value <0.05 was considered statistically significant. The results are presented as the mean ± SD. The supervised discriminant analysis was performed for the FA data to assess how clearly the sample types and diagnosis groups differed from each another, which individual FAs separated them most clearly, and how well the analysis was able to classify the samples to respective joint groups.

**Table 1:** Original *p* values from the generalized linear model (GLM) of the synovial fluid (SF) and its extracellular vesicle-enriched pellet (EV) fatty acids (FAs) and the calculated Benjamini–Hochberg (B–H) critical values used to control the false discovery rate

SF FA	***p*** group	SF FA	***p*** group × age	SF B–H critical value	EV FA	***p*** group	EV FA	***p*** group × age	EV B–H critical value
n-3/n-6 PUFAs	3 × 10^–14^*	n-3/n-6 PUFAs	3 × 10^–12^*	0.0005	18:0	0.002	22:0	0.002	0.0006
16:1n-7	7 × 10^–11^*	16:1n-7	1 × 10^–8^*	0.0011	22:0	0.003	18:0	0.004	0.0011
17:0*i*	3 × 10^–8^*	17:0*i*	1.5 × 10^–7^*	0.0016	16:0	0.020	16:1n-7	0.032	0.0017
Prod/prec n-6	0.00008*	Prod/prec n-6	0.0005*	0.0022	∆5-DI n-6	0.040	16:0	0.077	0.0022
18:2n-6	0.0006*	16:0*i*	0.0024*	0.0027	22:1n-9	0.040	22:1n-9	0.079	0.0028
18:0*i*	0.001*	18:0*i*	0.0031*	0.0033	20:4n-6	0.088	18:2n-6	0.097	0.0033
16:0*i*	0.001*	18:2n-6	0.0037*	0.0038	16:1n-9	0.091	18:3n-3	0.109	0.0039
∆5-DI n-6	0.003*	24:1n-9	0.0039*	0.0043	16:1n-7	0.093	Prod/prec n-3	0.139	0.0044
14:0	0.003*	22:0	0.0047*	0.0049	17:0*i*	0.108	15:0	0.162	0.0050
n-6 PUFAs	0.004*	∆5-DI n-6	0.010	0.0054	15:0	0.109	16:1n-9	0.187	0.0056
22:0	0.004*	n-6 PUFAs	0.012	0.0060	18:2n-6	0.121	∆5-DI n-6	0.202	0.0061
DMA 18:0	0.005*	DMA 18:0	0.015	0.0065	20:0	0.130	20:0	0.205	0.0067
24:1n-9	0.013	20:3n-6	0.020	0.0071	DMA 18:0	0.132	DBI	0.222	0.0072
∆6-DI n-6	0.019	n-3 PUFAs	0.029	0.0076	18:3n-3	0.133	SFAs	0.284	0.0078
n-3 PUFAs	0.023	14:0	0.029	0.0082	DMAs	0.172	UFAs/SFAs	0.312	0.0083
18:3n-6	0.032	18:3n-6	0.046	0.0087	24:0	0.198	DMAs	0.355	0.0089
20:3n-6	0.033	Prod/prec n-3	0.054	0.0092	Prod/prec n-3	0.199	17:0*ai*	0.412	0.0094
17:0*ai*	0.043	22:5n-3	0.054	0.0098	DBI	0.208	20:4n-6	0.437	0.0100

The *p* values that remained significant after the procedure were obtained by comparing the original *p* value to the corresponding critical value on the same row. Asterisk indicates comparisons for which the direction of the difference is confidently interpreted at the *α*/2 level. The SF FAs that originally had *p* values >0.05 in the GLM were excluded from the table. In the EV fraction, no significant differences remained after the procedure, but a similar number of FAs is shown

*ai anteiso*-methyl-branch, *DBI* double bond index, *DI* desaturation index, *DMA* dimethyl acetal, *i iso*-methyl-branch, *Prod/prec* product/precursor ratio, *PUFA* polyunsaturated fatty acid, *SFA* saturated fatty acid, *UFA* unsaturated fatty acid

## Results

### General variables

There were no statistically significant differences in the sex ratios (control: 5 mares, 3 geldings; CL/OA: 3 mares, 5 geldings; Fisherʼs exact test, *p* = 0.670) or average ages between the study groups (control: 9 ± 3.2 years; CL/OA: 12 ± 4.8 years; GLM, *p* = 0.182). However, the average body masses of the OA/CL horses were higher than those of the control group (control: 491 ± 65 kg; CL/OA: 544 ± 39 kg; GLM, *p* = 0.029). The control and CL joints showed significantly lower scores in OA grading than the OA joints (GLM, Fisherʼs exact test, *p* < 0.0005 for both).

### Differences in the FA profiles between sample types

**Fig. 1:**
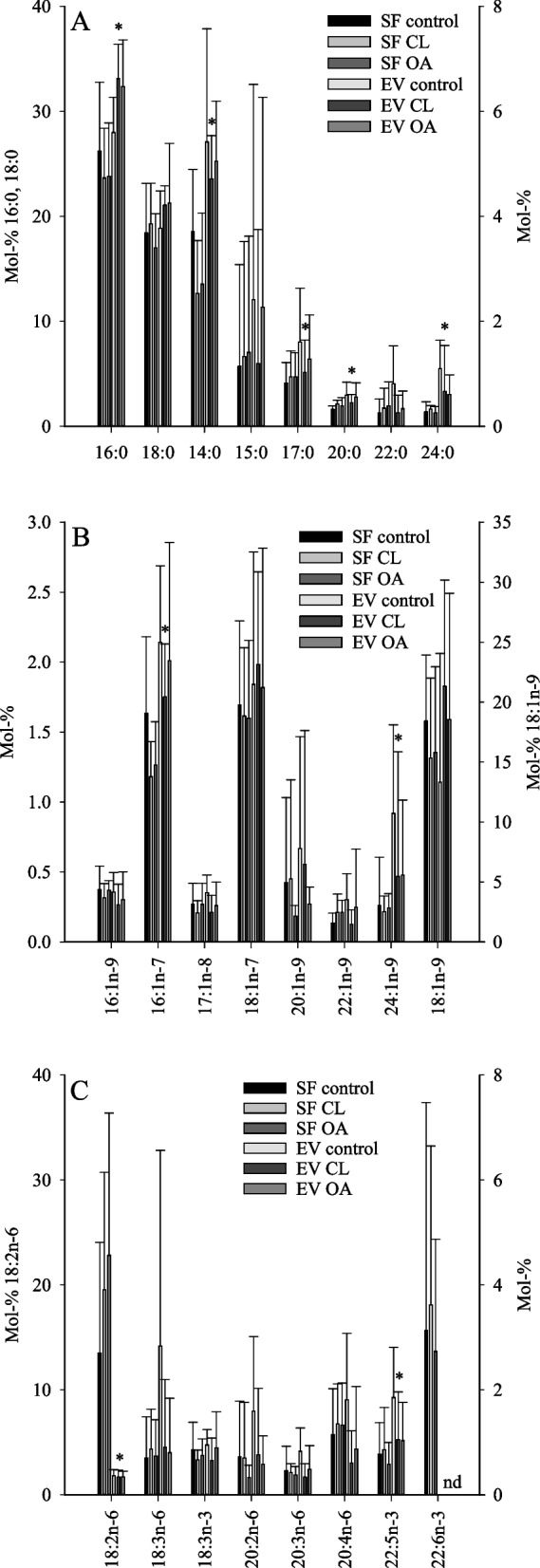
The proportions (mol-%) of selected saturated fatty acids (FAs) (panel **A**), monounsaturated FAs (panel **B**), and polyunsaturated FAs (panel **C**) in the equine synovial fluid (SF) and its ultracentrifuged extracellular vesicle-enriched pellet (EV) (mean + SD, *n* = 8 per sample group). Note that in panel **A**, 16:0 and 18:0 values are expressed on the left *y*-axis and the values of other FAs on the right *y*-axis. In panel **B**, 18:1n-9 values are expressed on the right *y*-axis and, in panel **C**, 18:2n-6 values are expressed on the left *y*-axis. SF 22:6n-3 also includes an unknown artefact. CL, contralateral; OA, osteoarthritis; nd, not determined. * = statistically significant difference between SF and EV (t-test, *p* < 0.05)

Compared to SFs, EV-enriched pellets had higher SFA sums and lower n-6 PUFA sums, when all diagnosis groups were pooled together (t-test, *p* < 0.0005; Table S1, S2). Regarding individual FAs, the average proportions of 14:0 (myristic acid), 16:0*i* (*i* = *iso-*methyl-branch), 16:0 (palmitic acid), 16:1n-7 (palmitoleic acid), 17:0*i*, 17:0, 18:0*i*, DMA 18:0, 18:1n-5, 20:0, 22:5n-3, 24:0 (lignoceric acid), and 24:1n-9 (nervonic acid) were higher in EV-enriched pellets compared to SFs, and the 18:2n-6 (linoleic acid) percentage was lower (t-test, *p* < 0.0005–0.045; Fig. 1, Table S1, S2). These comparisons were not performed for 22:6n-3 or any sums or indices containing it, as 22:6n-3 could not be determined in the EV fraction due to the presence of a large coeluting artefact in the same chromatographic peak.

### Effects of OA on the FA profiles of horse SF

The FA modifications that separated equine OA from control samples in the GLM included, for instance, elevated proportions of 18:2n-6 and total n-6 PUFAs and reduced percentages of 14:0, 16:1n-7, and 17:0*ai* (*ai* = *anteiso*-methyl-branch) (Fig. 1, Table S1). In addition, n-3/n-6 PUFA ratios and product/precursor ratios of n-6 PUFAs were lower in OA SF. All these variables, except of 17:0*ai*, remained significant after the Benjamini–Hochberg procedure (Table 1).

**Fig. 2:**
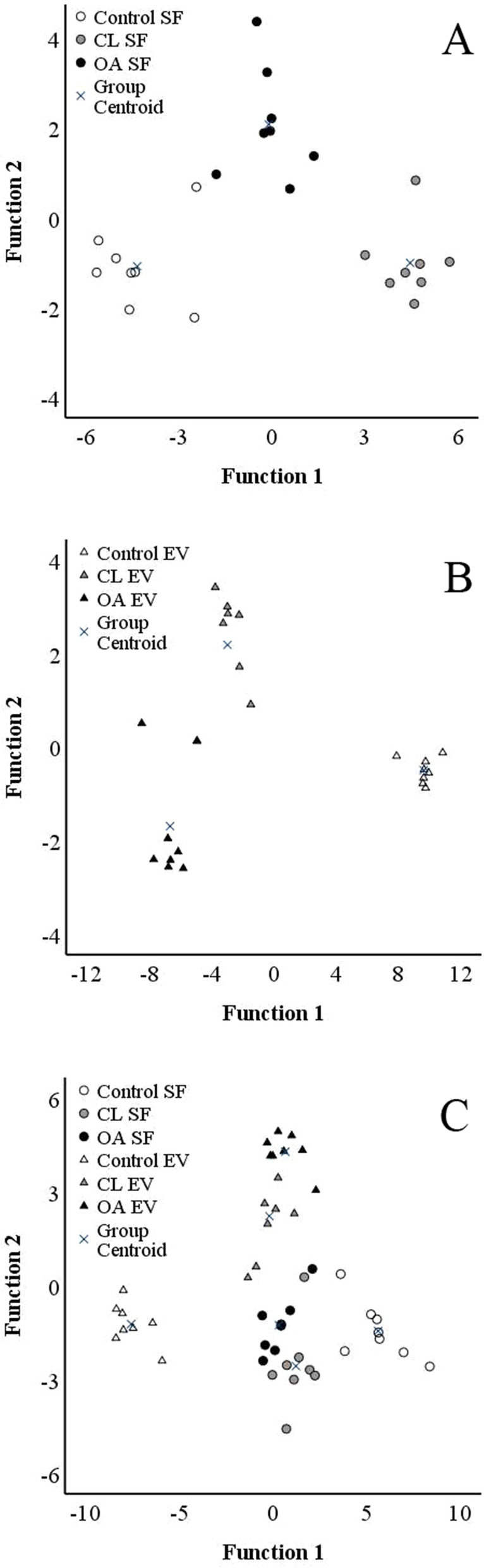
Discriminant analyses depicting the classification of fatty acid (FA) data in equine synovial fluid (SF) (panel **A**) and its ultracentrifuged extracellular vesicle-enriched pellet (EV) (panel **B**), and combined data (panel **C**) from control, contralateral (CL), and osteoarthritic (OA) fetlock joints based on discriminant functions 1 (on *x*-axis) and 2 (on *y*-axis). For each sample group, *n* = 8. In panel **A**, 16:1n-7, 20:0, 16:0*i*, 18:3n-3, and DMA 16:0 contributed to function 1, and 18:2n-6, 20:1n-9, 18:0, 20:2n-6, and 22:5n-3 contributed to function 2 with the highest separation power. In panel **B**, 22:5n-3, 18:3n-6, 20:2n-6, 24:0, and 18:0 contributed to function 1, and DMA 18:0, 17:0*i*, 20:3n-6, 18:3n-3, 20:0, and 16:0 to function 2, making them the most discriminative FAs between the diagnosis groups. In panel **C**, 22:0 (function 1), 16:0 (function 2), 18:2n-6, 17:0*ai*, 14:0, 17:0*i*, and 16:1n-7 (the last 5 in function 3) had the highest separation power

In the discriminant analysis, all joint groups aligned separately from each other based on the FA data (Fig. 2A). The most important contributors to the model included 16:1n-7, 20:0, 16:0*i*, 18:3n-3 (*α*-linolenic acid), and DMA 16:0 for discriminant function 1 on the *x*-axis, and 18:2n-6, 20:1n-9, 18:0 (stearic acid), 20:2n-6, and 22:5n-3 for discriminant function 2 on the *y*-axis. Function 1 explained 85.6% of the variance in the dataset and separated especially control SF from CL SF. Function 2 accounted for 14.4% of the variance and separated OA SF from control and CL SFs. The model classified 100% of the SF samples to their correct diagnosis group.

In control joints, the percentages of 16:0*i*, 16:1n-7, 17:0*i*, 18:0*i*, DMA 18:0, 18:3n-6, 20:3n-6 (dihomo-*γ*-linolenic acid), and 24:1n-9, n-3 PUFA sums, and n-3/n-6 PUFA ratios had an inverse association with age. The relation was positive for 22:0 (behenic acid) proportions and n-3/n-6 PUFA ratios in CL joints and for 14:0, 16:1n-7, and n-3/n-6 PUFA ratios in OA joints. 18:2n-6 and n-6 PUFA sum had an inverse association with age in OA joints. When all diagnosis groups were pooled together, there were no significant correlations in FA percentages, sums, ratios, or indices with age.

### Effects of OA on the FA profiles of SF EV-enriched pellets

The variables that separated equine OA from control samples in the GLM included 16:0, 18:0 (increased), and 22:0 (decreased) (Fig. 1A, Table S2), but the differences did not remain significant after controlling for multiple hypothesis testing (Table 1).

All study groups were clustered separately in the discriminant analysis (Fig. 2B). Function 1 that separated control EV-enriched pellets from those of the other groups explained 94.9% of the variance in the dataset, and function 2 separating CL and OA EV-enriched pellets accounted for 5.1% of the variance. For function 1, 22:5n-3, 18:3n-6, 20:2n-6, 24:0, and 18:0 were the most discriminative FAs between the diagnosis groups. The corresponding FAs for function 2 included DMA 18:0, 17:0*i*, 20:3n-6, 18:3n-3, 20:0, and 16:0. The analysis classified 95.8% of the EV-enriched pellets correctly to their respective diagnosis group, as one CL joint was misclassified among OA joints.

When the six study groups were analysed together by the discriminant analysis, function 1 (explaining 57.9% of the variance) separated especially control SFs from control EV-enriched pellets, whereas function 2 (22.5%) separated CL and OA SFs from CL and OA EV-enriched pellets (Fig. 2C). Overall, 95.8% of the samples were classified to the correct study group. One CL EV-enriched pellet was misclassified among OA SFs and another one clustered among OA EV-enriched pellets. The FAs 22:0 (function 1), 16:0 (function 2), 18:2n-6, 17:0*ai*, 14:0, 17:0*i*, and 16:1n-7 (function 3) had the highest separation power.

16:1n-7 in EV-enriched pellets showed a positive association with age in OA joints, whereas the relation was negative for 18:0 in OA joints and 22:0 in control joints. 22:0 also had an inverse correlation with age when all EV samples were pooled together (*r*_*s*_ = –0.419, *p* = 0.042).

## Discussion

The results of the present study demonstrate that joints with naturally occurring equine OA can be distinguished from normal joints based on their FA fingerprints in SF and its ultracentrifuged EV-enriched pellet. According to the univariate and multivariate analyses of the SF data, 18:2n-6 (increased), 14:0, and 16:1n-7 (decreased) were among the factors that discriminated OA joints from control samples and, regarding EV-enriched pellets, 16:0, 18:0 (increased), and 22:0 (decreased) belonged to the biomarkers of OA. To the best of our knowledge, this is the first time the detailed FAS of OA EVs has been reported in the horse. There are high hopes for the future that the horse could be utilized as a translational model to study EV-based therapies for joint diseases, since this athletic species develops primary OA like humans but provides substantially larger sample volumes.

It should be emphasized that even though the supervised discriminant analysis classified 95.8–100% of the samples to the correct diagnosis group, the predictive power of the unsupervised leave-one-out approach of the discriminant analysis (without prior assignment of samples to respective diagnostic groups) would be inadequate to diagnose equine OA based solely on FA profiles (data not shown). The diagnostic use of FAs was not the principal goal of this study that aimed to assess, which FAs would characterize OA joints and be the most likely ones to affect OA pathogenesis in either a beneficial or harmful manner. However, FA profiling could provide tools to identify susceptible individuals before overt symptoms arise, and FA manipulations could be utilized to prevent or slow down disease progression into debilitating OA.

### SF FA profiles of OA horses

An interesting finding regarding equine OA SF was the elevation in 18:2n-6, which is a dietarily essential PUFA mainly obtained from vegetable oils, nuts, and seeds [22]. Regarding the diet of stabled horses, hay and oats are rich sources of 18:2n-6 [23, 24]. The proportional increase from 13.5 to 22.8% can be considered biologically significant, and it was also reflected in the n-6 PUFA sums, product/precursor ratios of n-6 PUFAs, and in the n-3/n-6 PUFA ratios. The generally high proportions of 18:2n-6 in equine SF compared to humans [25] could relate to the herbivorous feeding habits of horses [26], albeit the difference was only detected for OA SFs. Previous literature reported increased accumulation of C18–22 n-6 PUFAs in the OA cartilage and bone of human patients [27–30], but the present data did not show elevated levels of C20 n-6 PUFAs. This could result from a low capacity to convert 18:2n-6 to its longer-chain derivatives in the horse [17], which may be further slowed down by OA, like previously proposed for human rheumatoid arthritis (RA) [31]. 18:2n-6 was reported to be the most abundant individual PUFA in the phosphatidylcholines (PCs) of equine SF, while the levels of PCs with 20:4n-6 (arachidonic acid) were clearly lower [17].

Previously in humans and mice, n-6 PUFAs in diet or circulation were positively associated with joint effusion, synovitis, and OA [32–35], and high plasma n-6/n-3 PUFA ratios associated with greater pain and functional limitations of knee OA [36]. In mouse and rabbit OA models, n-3/n-6 PUFA ratios in serum correlated negatively with OA severity [34] and reduced in the infrapatellar fat pad of OA knees [37], also supporting the present results. 18:2n-6 can induce potentially adverse effects on joint tissues, for instance, by stimulating the secretion of inflammatory agents and cartilage-degrading proteinases [38, 39]. Its effects can be mediated via eicosanoids, such as prostaglandin E_2_ [40], or pro-resolving lipid mediators including lipoxin A_4_ (LXA_4_) [41], both synthesized from 20:4n-6. Together with LXA_4_, 20:3n-6-derived prostaglandin E_1_ can induce anti-inflammatory effects on joints [22]. Thus, 18:2n-6 could stimulate resolution pathways via lipid mediator synthesis and, in addition to inflammation and resolution, it may also be able to affect cell proliferation and EV secretion [42, 43].

The proportions of 14:0 decreased in equine OA SF, whereas in human patients its levels were elevated in late-stage compared to early-stage OA [44]. 14:0 has potential to induce interleukin-6 secretion and glycosaminoglycan release from chondrocytes and/or RA synoviocytes [38, 45] and to inhibit bone resorption [46] but, compared to 16:0 and 18:0, it can also induce protective effects on cartilage integrity and joint health [45, 47, 48]. The observed reduction in the proportions of 16:1n-7 in CL and OA SFs supports earlier results from an OA mouse model with an inverse association between serum/SF 16:1n-7 and OA severity [34]. However, OA SF, cartilage, and bone have previously manifested elevated 16:1n-7 levels in humans [28, 29, 44]. The physiological roles of 16:1n-7 remain insufficiently understood, but there are indications that it may be anti-inflammatory and have beneficial effects on insulin sensitivity [49]. In chondrocytes and RA synoviocytes, 16:1n-7 was able to stimulate interleukin-6 secretion [38], but it can also reduce bone resorption [46].

FAs are associated with ageing-related diseases, and alterations in circulating FA levels have been documented with advancing age [50]. Even though OA is clearly age-dependent in humans, the relationship is less straightforward in horses that can develop signs of OA early in life [21, 51]. The present study examined the associations between the FA proportions and age of horses. Regarding SF, 14:0, 16:1n-7, and n-3/n-6 PUFA ratios were positively associated with advancing age in OA joints, while the relation was negative for 18:2n-6 and total n-6 PUFAs. This could indicate, for instance, that the OA-induced increases in 18:2n-6 levels were less pronounced in older animals. In control SF, the associations were negative for 18:3n-6, 20:3n-6, total n-3 PUFAs, and n-3/n-6 PUFA ratios. In addition to oxygenase enzymes, n-3 and n-6 PUFAs are known to compete for the same desaturases [52], the activities of which can change with advancing age [53]. It seems plausible that OA could intervene the complicated age-related modifications in PUFA metabolism. In humans, FA chain length (either increased or decreased) and double bond index (decreased) have been reported to change in response to OA with potential influence on the lubricating properties of SF [20, 54, 55]. In the present study, total average chain length and double bond index remained unaffected by equine OA, and we did not observe increases in long-chain SFAs or MUFAs occasionally associated with OA [56].

The *post-mortem* examination of the MCP joint surfaces revealed that most of the CL joints had mild-to-moderate OA. This was also reflected in their FA composition with, for instance, reduced proportions of 16:1n-7, 17:0*ai* (SF), and 22:0 (EV-enriched pellet). Figure 2C on the discriminant analysis visualizes how CL samples clustered close to OA joints clearly separately from control samples. This strongly supports the notion that the CL joints of OA animals cannot be routinely considered healthy controls, nor their SF used for lipidomic research without a proper control group [37]. In fact, CL joints could be interesting research subjects *per se*, to determine if the processes that cause OA are also affecting the CL joint but at a slower rate, and if the altered gait and possible favouring of the less symptomatic limb cause the observed biochemical changes through increased loading. In humans, unilateral knee OA is a well-known risk factor for the development of bilateral disease [57], and the possibility that equine OA would be a symmetrical disease should also be investigated further [58, 59].

### FA profiles in the SF EV-enriched pellets of OA horses

The general differences between sample types were featured by higher SFA sums and lower n-6 PUFA sums in EV-enriched pellets compared to SFs. Regarding individual FAs of biological interest, SFAs 16:0 and 24:0 and MUFAs 16:1n-7 and 24:1n-9 were elevated, while 18:2n-6 was reduced in EV-enriched pellets. Arthritic human SF contains neutrophils, monocytes, and T cells [60] that presumably are among the cellular sources of EVs in OA SF. In addition to leukocytes, fibroblast-like synoviocytes (FLSs) and chondrocytes represent other cell types in synovial joints that are known to secrete EVs [11]. It could be hypothesized that in the course of synovitis, the proportion of EVs released, for instance, by neutrophils would increase [7], and the SF EV population would increasingly represent this cellular origin in its FAS. We previously observed in the same horse population that naturally occurring OA did not affect the numbers of small EVs, but there was an inverse correlation between the OA grade and the count of large EVs [19]. Furthermore, OA and CL SFs had a reduced number and % of large EVs that transport hyaluronic acid.

Lipids have structural roles in EVs and affect their membrane stability, EV binding and uptake to recipient cells, and the formation and release of EVs [12, 61]. FA components also provide precursors for the biosynthesis of lipid mediators [62]. Regarding synovial joints, EVs have been shown to enter, for instance, FLSs and chondrocytes, and to modify their gene expression [63, 64]. Hypothetically, EVs could contribute to decreased cell survival and increased production of inflammatory and cartilage-degrading factors in OA joints. The present study documented elevated proportions of 16:0 and 18:0 in EV-enriched pellets from equine OA SF. In general, dietary SFAs, such as 16:0 and 18:0, have been associated with obesity, inflammation, and the development of OA [65]. Regarding arthritis, they can have adverse effects on cartilage degradation, induce subchondral bone changes, and affect pain perception [45, 48]. SFAs are important constituents of EVs secreted by FLSs [66], but their increased proportions in OA EVs could be considered undesirable, as they can have pro-inflammatory and catabolic effects on joint tissues [45, 67]. It should, however, be recalled that the FAs analysed in the present study mainly originated from esterified lipids, while the effects of individual FAs on joint health have often been investigated with non-esterified FAs.

When chondrocytes/cartilage explants of human, bovine, and murine origin were treated with 16:0 or 18:0, the production of inflammatory agents increased together with stimulated apoptosis, endoplasmic reticulum stress, and extracellular matrix degradation [45, 67–70]. The elevated secretion or expression of pro-inflammatory factors (interleukins, monocyte chemoattractant protein-1, cyclooxygenase-2) was also observed in synoviocytes treated with 16:0 (or 18:0) [38, 68]. Regarding bone, these SFAs can stimulate resorption, reduce bone formation, induce the production of inflammatory agents, and decrease the mineral density of subchondral bone [39, 45, 46]. While previous studies have reported potentially positive effects of EVs from SF and MSCs on equine chondrocytes [13–15], the present results suggest that OA SF could contain EVs with a pro-inflammatory FA profile. In fact, there are indications from C2C12 myoblasts that 16:0-enriched EVs are able to transfer the effects of 16:0 to neighbouring cells [71]. Our results are different from equine asthma, the severe form of which shows a loss of 16:0 in EVs isolated from bronchoalveolar lavage fluid [Höglund et al., unpubl. data]. In contrast to 16:0, the proportions of 22:0 decreased in EV-enriched pellets from CL and OA joints, and it also showed a negative association with age, especially in control joints. 22:0 is a minor, long-chain SFA that has also previously been detected in EV lipids [72], but its physiological role in inflammatory diseases remains unravelled.

There are some limitations in the present study that should be considered. The SF samples were not centrifuged before freezing to remove cells and debris. The EV yield by ultracentrifugation may have been limited due to the lack of hyaluronidase pre-treatment of the SF samples [13]. The enrichment of EVs by ultracentrifugation can result in the co-isolation of EVs with lipoproteins that contain triacylglycerols and cholesteryl esters instead of membrane lipids [73]. In that scenario, the FA analysis of the EV fraction may have been affected by some remnant lipids, while cell organelles and protein aggregates could have also contaminated the ultracentrifuged fraction. The SF apolipoprotein B (and PL) levels of horses are known to be significantly lower than those of humans when examined from healthy joints [18]. However, this is not necessarily the case for OA SFs, as the permeability of synovial membrane may increase due to inflammation [74]. Moreover, 18:2n-6 and 18:1n-9 that are important constituents of horse SF PCs would have been expected to increase in proportion in addition to 16:0 and 18:0 [17], if SF lipoproteins had been a significant confounding factor in the FA analysis. The increased levels of 24:0 and 24:1n-9 in EV-enriched pellets *vs.* SFs could indicate that the isolation procedure had worked properly, since these FAs are often abundant in EV membrane sphingolipids [12, 73]. Overall, the results regarding EV-enriched pellets must be taken with some caution, as the Benjamini–Hochberg procedure did not accept these differences as significant.

To conclude, naturally occurring equine OA causes alterations in the FA signatures of SF and its EV-enriched pellet, and these OA-specific changes may induce pro-inflammatory and catabolic effects on joint tissues. Most prominently, the potentially harmful 18:2n-6 increases in proportion in the SF of OA joints indicating its significance as both a biomarker and a potential effector in disease progression. In arthritic EVs, elevated 16:0 and 18:0 could be useful for further study regarding translational research. In addition, the large volume of SF in equine joints makes them attractive targets for detailed analyses of SF biochemistry before and after interventions, which can eventually lead to new therapeutic approaches regarding both EV-based treatment options and manipulations of the SF FA profile. Approaching equine OA from the perspective of lipids holds many future possibilities in veterinary practice and may lead to potentially valuable applications in human OA.

## Supplementary Information

**Additional file 1: Table S1.** Fatty acid profiles (mol-%) of equine synovial fluids according to diagnosis (mean ± SD, n = 8 for each sample group).

**Additional file 2: Table S2.** Fatty acid profiles (mol-%) of equine synovial fluid extracellular vesicle-enriched pellets according to diagnosis (mean ± SD, n = 8 for each sample group).

## Data Availability

The raw dataset that was generated and used for analyses during the current study is available at https://studentuef-my.sharepoint.com/:b:/g/personal/tpniemin_uef_fi/EeSh8oW3KwhOhs-i0yM93vYBG0f_1gA__7_jueX04HWy_w?e=VMPlff and by request from the corresponding author.

## Abbreviations

CL: Contralateral
DMA: Dimethyl acetal
DPBS: Dulbeccoʼs phosphate buffered saline
EV: Extracellular vesicle
FA: Fatty acid
FAS: Fatty acid signature
FLS: Fibroblast-like synoviocyte
GLM: Generalized linear model
LXA_4_: Lipoxin A_4_
MCP: Metacarpophalangeal
MSC: Mesenchymal stem cell
MUFA: Monounsaturated fatty acid
OA: Osteoarthritis
PC: Phosphatidylcholine
PL: Phospholipid
PUFA: Polyunsaturated fatty acid
RA: Rheumatoid arthritis
r_s_: Spearman correlation coefficient
SF: Synovial fluid
SFA: Saturated fatty acid
