# Supplementary material for: Equine osteoarthritis modifies fatty acid signatures in synovial fluid and its extracellular vesicles
Source: Arthritis Res Ther. 2023 Mar 9;25:39. doi: 10.1186/s13075-023-02998-9 (PMC9996872; doi:10.1186/s13075-023-02998-9)
Supplement: Supplementary file 1 — Additional file 1: Table S1. Fatty acid profiles (mol-%) of equine synovial fluids according to diagnosis (mean ± SD, n = 8 for each sample group). [file 13075_2023_2998_MOESM1_ESM.pdf]

Table S1. Fatty acid profiles (mol-%) of equine synovial fluids according to diagnosis (mean ± SD, n = 8 for each sample group).

Fatty acid	Control	Contralateral	Osteoarthritis	*p* group[a]	*p* group × age[a]
14:0	3.706 ± 1.191	2.529 ± 1.010	2.708 ± 1.354	0.003*	0.029
15:0	1.145 ± 1.934	1.329 ± 2.193	1.408 ± 2.214	0.641	0.449
16:0*i*	0.557 ± 0.423	0.358 ± 0.111	0.516 ± 0.163	0.001*	0.002*
DMA 16:0	0.488 ± 0.177	0.605 ± 0.328	0.553 ± 0.257	0.585	0.407
16:0	26.226 ± 6.545	23.660 ± 4.743	23.836 ± 5.064	0.625	0.337
16:1n-9	0.376 ± 0.167	0.316 ± 0.100	0.370 ± 0.070	0.342	0.450
16:1n-7	1.635 ± 0.547	1.181 ± 0.250	1.265 ± 0.309	<0.0005*	<0.0005*
17:0*i*	0.831 ± 0.592	0.694 ± 0.194	0.608 ± 0.236	<0.0005*	<0.0005*
17:0*ai*	0.362 ± 0.160	0.188 ± 0.073	0.246 ± 0.089	0.043	0.477
17:0	0.825 ± 0.390	0.941 ± 0.492	0.943 ± 0.459	0.671	0.537
17:1n-8	0.272 ± 0.147	0.208 ± 0.087	0.268 ± 0.150	0.192	0.352
18:0*i*	0.892 ± 0.712	0.777 ± 0.534	0.756 ± 0.528	0.001*	0.003*
DMA 18:0	0.283 ± 0.215	0.251 ± 0.063	0.224 ± 0.118	0.005*	0.015
18:0	18.419 ± 4.738	19.273 ± 3.889	16.981 ± 3.286	0.655	0.430
18:1n-9	18.418 ± 5.512	15.334 ± 6.680	15.791 ± 7.155	0.180	0.130
18:1n-7	1.695 ± 0.600	1.615 ± 0.488	1.600 ± 0.557	0.840	0.452
18:1n-5	0.862 ± 0.556	0.731 ± 0.430	0.571 ± 0.429	0.085	0.285
18:2n-6	13.529 ± 10.528	19.541 ± 11.195	22.828 ± 13.563	0.001*	0.004*
18:3n-6	0.702 ± 0.786	0.871 ± 0.763	0.743 ± 0.687	0.032	0.046
18:3n-3	0.862 ± 0.521	0.667 ± 0.187	0.749 ± 0.316	0.211	0.277
20:0	0.324 ± 0.067	0.423 ± 0.071	0.393 ± 0.153	0.161	0.628
20:1n-9	0.426 ± 0.606	0.451 ± 0.708	0.184 ± 0.076	0.606	0.646
20:2n-6	0.721 ± 1.064	0.701 ± 1.062	0.331 ± 0.233	0.134	0.097
20:3n-6	0.465 ± 0.460	0.423 ± 0.171	0.379 ± 0.160	0.033	0.020
20:4n-6	1.145 ± 0.877	1.352 ± 0.763	1.326 ± 0.803	0.663	0.417
22:0	0.257 ± 0.260	0.347 ± 0.380	0.391 ± 0.454	0.004*	0.005*
22:1n-9	0.136 ± 0.071	0.211 ± 0.131	0.211 ± 0.087	0.244	0.695
22:5n-3	0.775 ± 0.599	0.862 ± 0.800	0.587 ± 0.409	0.066	0.054
22:6n-3 w/ artefact	3.130 ± 4.350	3.622 ± 3.026	2.734 ± 2.136	0.095	0.101
24:0	0.275 ± 0.194	0.325 ± 0.071	0.257 ± 0.121	0.070	0.084
24:1n-9	0.261 ± 0.344	0.215 ± 0.113	0.243 ± 0.103	0.013	0.004*
Σ:SFA	53.820 ± 9.266	50.843 ± 6.947	49.041 ± 8.955	0.265	0.321
Σ:MUFA	24.080 ± 5.924	20.263 ± 7.943	20.504 ± 8.103	0.100	0.125
Σ:PUFA	21.330 ± 13.479	28.039 ± 12.662	29.677 ± 15.088	0.089	0.113
Σ:n-6 PUFA	16.563 ± 10.863	22.887 ± 11.364	25.607 ± 14.060	0.004*	0.012
Σ:n-3 PUFA	4.767 ± 4.698	5.152 ± 3.208	4.070 ± 2.035	0.023	0.029
UFA/SFA	0.907 ± 0.429	0.982 ± 0.270	1.086 ± 0.393	0.218	0.350
n-3/n-6 PUFA	0.310 ± 0.215	0.245 ± 0.122	0.201 ± 0.105	<0.0005*	<0.0005*
Σ:DMA	0.770 ± 0.340	0.856 ± 0.359	0.778 ± 0.275	0.898	0.437
Δ9-DI	0.527 ± 0.111	0.450 ± 0.144	0.474 ± 0.138	0.080	0.162
Δ6-DI n-6 PUFA	0.096 ± 0.128	0.074 ± 0.083	0.065 ± 0.090	0.019	0.060
Δ5-DI n-6 PUFA	3.037 ± 1.462	3.181 ± 1.517	3.372 ± 1.432	0.003*	0.010
Prod/prec n-6 PUFA	0.151 ± 0.109	0.119 ± 0.068	0.083 ± 0.020	<0.0005*	0.001*
Prod/prec n-3 PUFA	4.017 ± 2.219	6.750 ± 4.744	5.010 ± 3.396	0.164	0.054
DBI	0.859 ± 0.388	0.981 ± 0.287	0.971 ± 0.296	0.631	0.428
TACL	17.346 ± 0.258	17.476 ± 0.201	17.408 ± 0.208	0.462	0.345

i = *iso*-methyl-branch, DMA = dimethyl acetal, *i.e.*, plasmalogen alkenyl chain derivative, *ai* = *anteiso*-methyl-branch, SFA = saturated fatty acid, MUFA = monounsaturated fatty acid, PUFA = polyunsaturated fatty acid, UFA = unsaturated fatty acid (MUFA + PUFA), DI = desaturation index, prod/prec = product/precursor ratio, DBI = double bond index, TACL = total average chain length, [a] = generalized linear model, * = significant differences were retained after the Benjamini–Hochberg procedure
