# Supplementary material for: Equine osteoarthritis modifies fatty acid signatures in synovial fluid and its extracellular vesicles
Source: Arthritis Res Ther. 2023 Mar 9;25:39. doi: 10.1186/s13075-023-02998-9 (PMC9996872; doi:10.1186/s13075-023-02998-9)
Supplement: Supplementary file 2 — Additional file 2: Table S2. Fatty acid profiles (mol-%) of equine synovial fluid extracellular vesicle-enriched pellets according to diagnosis (mean ± SD, n = 8 for each sample group). [file 13075_2023_2998_MOESM2_ESM.pdf]

Table S2. Fatty acid profiles (mol-%) of equine synovial fluid extracellular vesicle-enriched pellets according to diagnosis (mean ± SD, n = 8 for each sample group).

**Fatty acid**	**Control**	**Contralateral**	**Osteoarthritis**	***p* group**[a]	***p* group × age**[a]
14:0	5.418 ± 2.159	4.715 ± 0.828	5.049 ± 1.144	0.576	0.854
15:0	2.416 ± 4.098	1.194 ± 2.556	2.271 ± 4.000	0.109	0.162
16:0*i*	1.765 ± 1.495	0.696 ± 0.724	1.015 ± 1.401	0.362	0.731
DMA 16:0	0.746 ± 0.312	0.408 ± 0.213	0.509 ± 0.414	0.482	0.539
16:0	27.997 ± 3.358	33.128 ± 3.294	32.391 ± 4.415	0.020	0.077
16:1n-9	0.357 ± 0.139	0.265 ± 0.148	0.303 ± 0.198	0.091	0.187
16:1n-7	2.142 ± 0.547	1.752 ± 0.379	2.012 ± 0.845	0.093	0.032
17:0*i*	2.331 ± 0.971	1.100 ± 0.653	1.362 ± 0.809	0.108	0.577
17:0*ai*	0.402 ± 0.211	0.297 ± 0.085	0.313 ± 0.093	0.812	0.412
17:0	1.604 ± 1.027	1.027 ± 0.618	1.282 ± 0.836	0.453	0.715
17:1n-8	0.353 ± 0.126	0.210 ± 0.122	0.259 ± 0.168	0.441	0.654
18:0*i*	2.196 ± 1.070	0.954 ± 0.771	1.046 ± 0.783	0.743	0.820
DMA 18:0	0.820 ± 0.356	0.273 ± 0.143	0.427 ± 0.339	0.132	0.474
18:0	18.869 ± 3.562	21.071 ± 1.851	21.261 ± 5.705	0.002	0.004
18:1n-9	13.309 ± 10.752	21.328 ± 8.855	18.569 ± 10.506	0.567	0.799
18:1n-7	1.841 ± 0.947	1.985 ± 0.660	1.819 ± 0.996	0.794	0.950
18:1n-5	1.362 ± 0.818	1.066 ± 0.426	1.223 ± 0.622	0.827	0.446
18:2n-6	1.801 ± 0.604	1.694 ± 0.657	1.716 ± 0.530	0.121	0.097
18:3n-6	2.838 ± 3.727	0.909 ± 1.291	0.804 ± 1.036	0.976	0.756
18:3n-3	0.949 ± 0.295	0.652 ± 0.430	0.899 ± 0.686	0.133	0.109
20:0	0.596 ± 0.242	0.448 ± 0.157	0.556 ± 0.274	0.130	0.205
20:1n-9	0.668 ± 0.799	0.553 ± 0.958	0.272 ± 0.120	0.854	0.822
20:2n-6	1.597 ± 1.420	0.760 ± 1.271	0.586 ± 0.536	0.707	0.958
20:3n-6	0.829 ± 0.446	0.339 ± 0.255	0.487 ± 0.451	0.379	0.766
20:4n-6	1.807 ± 1.273	0.605 ± 0.615	0.874 ± 1.190	0.088	0.437
22:0	0.813 ± 0.720	0.259 ± 0.331	0.335 ± 0.337	0.003	0.002
22:1n-9	0.303 ± 0.184	0.126 ± 0.103	0.248 ± 0.417	0.040	0.079
22:5n-3	1.853 ± 0.959	1.056 ± 0.905	1.032 ± 0.732	0.306	0.821
22:6n-3	nd	nd	nd	nd	nd
24:0	1.099 ± 0.540	0.664 ± 0.875	0.602 ± 0.377	0.198	0.515
24:1n-9	0.919 ± 0.633	0.467 ± 0.891	0.478 ± 0.536	0.337	0.539
Σ:SFA	65.505 ± 10.577	65.553 ± 7.041	67.483 ± 10.539	0.449	0.284
Σ:MUFA	21.255 ± 11.254	27.752 ± 8.299	25.182 ± 10.925	0.614	0.694
Σ:PUFA	11.673 ± 4.887	6.014 ± 4.094	6.398 ± 3.857	0.279	0.643
Σ:n-6 PUFA	8.871 ± 4.114	4.307 ± 3.037	4.467 ± 2.712	0.373	0.692
Σ:n-3 PUFA	2.802 ± 1.222	1.708 ± 1.130	1.931 ± 1.388	0.214	0.519
UFA/SFA	0.537 ± 0.248	0.529 ± 0.151	0.495 ± 0.203	0.575	0.312
n-3/n-6 PUFA	0.362 ± 0.174	0.410 ± 0.132	0.444 ± 0.287	0.781	0.859
Σ:DMA	1.566 ± 0.451	0.681 ± 0.328	0.935 ± 0.710	0.172	0.355
Δ9-DI	0.438 ± 0.253	0.497 ± 0.163	0.466 ± 0.215	0.610	0.452
Δ6-DI n-6 PUFA	2.420 ± 4.259	0.773 ± 1.482	0.454 ± 0.464	0.982	0.854
Δ5-DI n-6 PUFA	2.058 ± 1.040	1.622 ± 0.487	1.577 ± 0.602	0.040	0.202
Prod/prec n-6 PUFA	1.474 ± 0.766	0.596 ± 0.504	0.808 ± 0.827	0.293	0.831
Prod/prec n-3 PUFA	1.881 ± 0.524	1.987 ± 1.361	1.343 ± 0.738	0.199	0.139
DBI	0.584 ± 0.158	0.461 ± 0.109	0.450 ± 0.141	0.208	0.222
TACL	17.087 ± 0.268	17.090 ± 0.110	17.000 ± 0.134	0.972	0.979

i = *iso*-methyl-branch, DMA = dimethyl acetal, *i.e.*, plasmalogen alkenyl chain derivative, *ai* = *anteiso*-methyl-branch, SFA = saturated fatty acid, MUFA = monounsaturated fatty acid, PUFA = polyunsaturated fatty acid, UFA = unsaturated fatty acid (MUFA + PUFA), DI = desaturation index, prod/prec = product/precursor ratio, DBI = double bond index, TACL = total average chain length, nd = not determined, [a] = generalized linear model, significant differences were not retained after the Benjamini–Hochberg procedure
